# Characterizing Endocrine Status, Tumor Hypoxia and Immunogenicity for Therapy Success in Epithelial Ovarian Cancer

**DOI:** 10.3389/fendo.2021.772349

**Published:** 2021-11-17

**Authors:** Madison Pereira, Kathy Matuszewska, Colin Jamieson, Jim Petrik

**Affiliations:** Department of Biomedical Sciences, University of Guelph, Guelph, ON, Canada

**Keywords:** ovarian cancer, hypoxia, tumor microenvironment, endocrine cancer, immunotherapy

## Abstract

Epithelial ovarian cancer is predominantly diagnosed at advanced stages which creates significant therapeutic challenges. As a result, the 5-year survival rate is low. Within ovarian cancer, significant tumor heterogeneity exists, and the tumor microenvironment is diverse. Tumor heterogeneity leads to diversity in therapy response within the tumor, which can lead to resistance or recurrence. Advancements in therapy development and tumor profiling have initiated a shift from a “one-size-fits-all” approach towards precision patient-based therapies. Here, we review aspects of ovarian tumor heterogeneity that facilitate tumorigenesis and contribute to treatment failure. These tumor characteristics should be considered when designing novel therapies or characterizing mechanisms of treatment resistance. Individual patients vary considerably in terms of age, fertility and contraceptive use which innately affects the endocrine milieu in the ovary. Similarly, individual tumors differ significantly in their immune profile, which can impact the efficacy of immunotherapies. Tumor size, presence of malignant ascites and vascular density further alters the tumor microenvironment, creating areas of significant hypoxia that is notorious for increasing tumorigenesis, resistance to standard of care therapies and promoting stemness and metastases. We further expand on strategies aimed at improving oxygenation status in tumors to dampen downstream effects of hypoxia and set the stage for better response to therapy.

## Epithelial Ovarian Cancer

Epithelial ovarian cancer (EOC) is the most lethal gynecological cancer, and it is the fifth leading cause of cancer related deaths in women (1). A lack of disease-specific symptoms makes early detection difficult, with most women being diagnosed with EOC at an advanced stage (2). At diagnosis, most women have a large primary ovarian tumor, multiple metastatic secondary tumors and abdominal ascites (3). With the advanced stage at diagnosis, it is difficult to effectively treat the disease resulting in a 5-year survival rate for women diagnosed at stages 3 and 4 of 42% and 26% respectively (4).

Ovarian cancer most commonly presents in post-menopausal women at which point there are other age-related physiologic changes. Contributing risk factors include a familial history of EOC, increased lifetime ovulatory events due to nulliparity, undergoing hormone replacement therapy (HRT) and comorbidity factors such as diabetes and obesity (5, 6). Many of these risk factors involve prolonged exposure to steroid hormones. While post-menopausal women experience a decrease in hormone production, prolonged and chronic exposure to these hormones throughout their life contribute to an increased risk of EOC (7). Similarly, post-menopausal women on HRT have a further increase in exposure, enhancing the risk of EOC (8).

Currently, the common treatment protocol for ovarian cancer includes cytoreductive surgical debulking accompanied with chemotherapy, typically carboplatin and paclitaxel (9). Although there is often initial responsiveness to chemotherapy, most women develop chemoresistance and disease recurrence (10). As such, novel therapeutic approaches are needed to prevent chemoresistance and improve treatment success.

## The Endocrine System in Ovarian Cancer Tumorigenesis

The ovary is surrounded by a single layer of epithelial cells called the ovarian surface epithelium (OSE). The activity of the OSE is hormone-dependent; the ovary is a primary endocrine organ where both peptide and steroid hormones act on the OSE cells to mediate their activity throughout various reproductive processes. These hormones have an influential role on proliferation and differentiation of OSE cells and aberrant endocrine signaling can inflate the risk of EOC and contribute to its tumorigenesis (11, 12).

Gonadotropin-releasing hormone (GnRH) is a peptide hormone secreted by the hypothalamus. Its primary function is to regulate the production and release of luteinizing hormone (LH) and follicle stimulating hormone (FSH) from the anterior pituitary (11). These hormones, with the addition of human chorionic gonadotrophin (hCG), have been shown to elicit pro-proliferative effects on EOC cells through activation of gonadotropin-response genes, increased growth factor signaling and ovarian production of sex steroids, suggesting an indirect role of GnRH in EOC tumorigenesis (13–15). Additionally, GnRH stimulation of EOC cell-bound GnRH Type I receptors elicit anti-proliferative and anti-apoptotic signals through mechanisms involving activation of phosphotyrosine phosphatase and NFκB, respectively (16, 17). At the level of the ovary, imbalances of inhibin and activin expression in tumor cells may further attribute to EOC tumorigenesis by supporting cell survival and stimulating proliferation (18). These proteins are secreted by the ovary and function primarily to regulate FSH production at the level of the anterior pituitary. Activin directly stimulates FSH from the anterior pituitary and inhibin suppresses activin signaling by binding and sequestering it thereby preventing it from binding to its receptors (11). Additional evidence has suggested paracrine and/or autocrine functions of these proteins in the ovarian tumor microenvironment (TME) with further influences on ovarian steroid synthesis, proliferation and tumor invasion (18–20) although the mechanisms by which these proteins influence these processes remain unclear.

The involvement of steroid hormones in ovarian cancer carcinogenesis is supported by both experimental and epidemiological findings (21, 22). Protective effects of oral contraceptives, particularly progestin-only formulations, as well as multiparity suggest an inverse relationship between progesterone and ovarian cancer risk (23, 24). Experimental findings on progesterone’s role in EOC are conflicting with both positive and negative effects on tumor invasiveness and metastasis (25, 26). Protective effects of breastfeeding and pregnancy suggest a similar inverse relationship between estrogen levels and EOC risk (11, 22). Experimental findings support a pro-tumorigenic role of estrogen and suggests estrogen may elicit EOC cell proliferation through increases in growth factor receptor expression, stimulation and/or increased expression of c-myc (27–29). Elevated risk associated with previous polycystic ovarian syndrome diagnosis and treatment with Danazol, a therapy used in the treatment of endometriosis, suggests a pro-tumorigenic role of androgens in EOC (22, 30). Elevated levels of dihydrotestosterone and testosterone are correlated with an increase in tumor volume in EOC and elevated dihydrotestosterone alone can increase IL-6 mRNA and protein levels and suppress the anti-proliferative effects of TGF-β1 resulting in EOC cell proliferation (31, 32).

**Figure 1:**
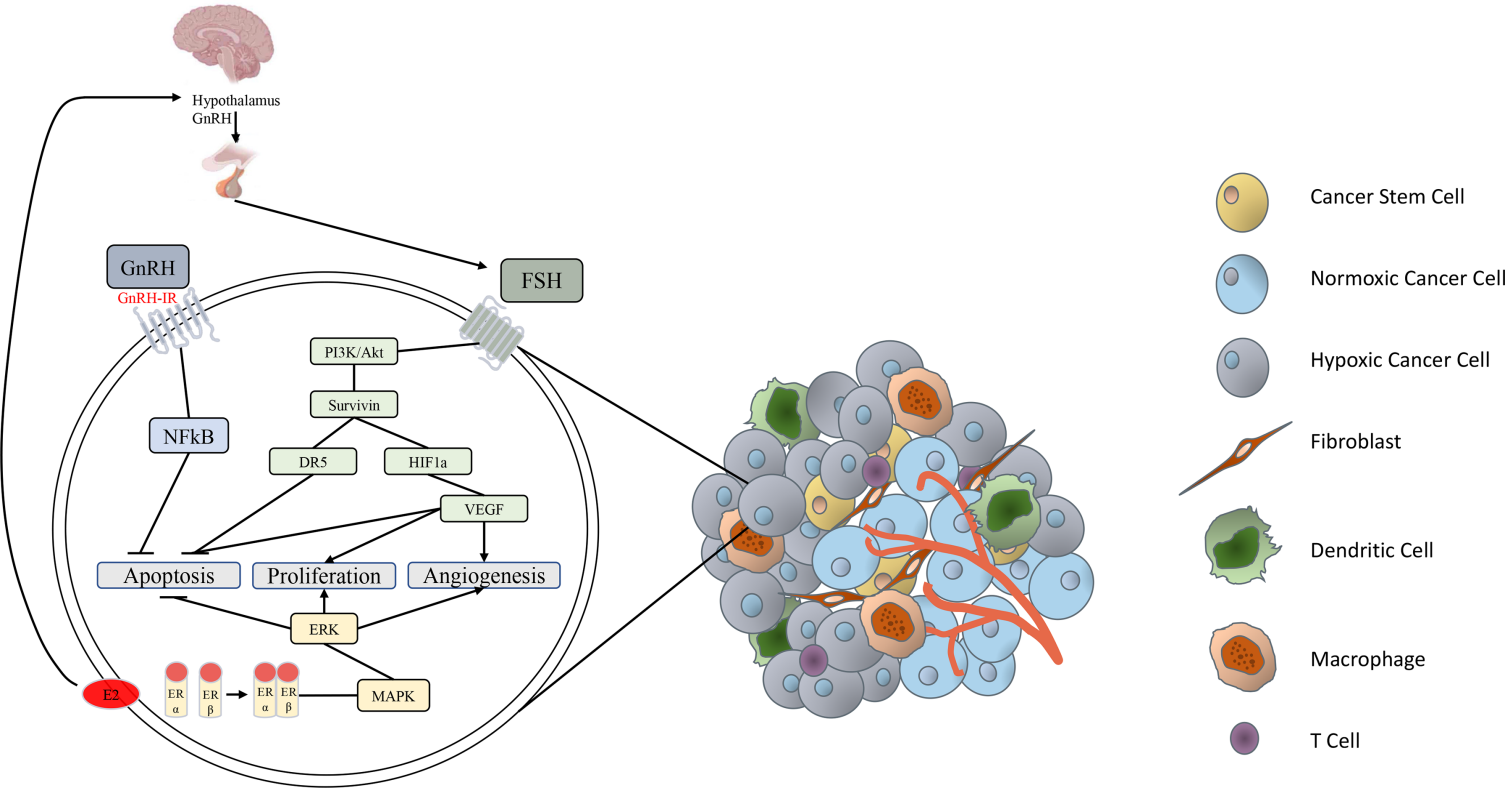
Simplified schematic of endocrine factors involved in epithelial ovarian cancer progression. GnRH secreted from the hypothalamus stimulates release of FSH from the anterior pituitary. FSH binds to the FSH receptor to activate the PI3K/Akt pathway, stimulating the expression of survivin (33). Survivin activates HIF-1α, resulting in secretion of VEGF, which inhibits apoptosis, stimulates proliferation and induces angiogenesis in various carcinomas (34). FSH-induced survivin upregulation is also associated with inhibition of death receptor DR5 and de-activation of an intracellular death-induced silencing complex (33). GnRH binding to the GnRH-I receptor activates intracellular NFkB, which inhibits ovarian cancer cell apoptosis (17, 35). Estrogen has direct effects by inducing activation of the proliferative, survival and angiogenic MAPK/ERK signaling pathway (36, 37). Estrogen also has an indirect pro-tumor effect by stimulating subsequent release of GnRH and FSH (38, 39).

With the growing body of epidemiological and experimental evidence, an association between the endocrine status of the patient and ovarian cancer tumorigenesis is well-supported as summarized in **Figure 1**. However, the specific mechanisms in which hormones impart their effects remain unclear. Future research is needed to elucidate the role of hormonal stimulation in EOC risk and progression.

## The Role of Angiogenesis in Ovarian Cancer

Angiogenesis is a naturally occurring process to develop new blood vessels from pre-existing vasculature. Angiogenesis is involved in a number of homeostatic processes including vascular repair in wound healing (40). In response to an injury, pro-angiogenetic stimulators including vascular endothelial growth factor (VEGF), fibroblast growth factor, platelet-derived growth factors, angiopoietins, hypoxia inducible factor (HIF) and many more are activated (41–45). When the wound has been repaired, anti-angiogenic factors including thrombospondin-1, endostatin and platelet factor 4 (46–48) are activated to counteract the pro-angiogenic stimulus. This switch inhibits further angiogenesis and prevents uncontrolled vessel formation. Although angiogenesis is generally quiescent in the adult, in the ovary, cyclical angiogenesis occurs and is an important process that helps regulate ovarian function (49). Perifollicular vascularization occurs during the ovarian cycle to support development and function of the growing follicles (50). Rapid angiogenesis occurs during initial formation of the corpus luteum (51). LH and prostaglandins can specifically trigger the activation of angiogenesis following initiation of ovulatory events to accelerate capillary formation within the follicle and developing corpus luteum (52). Similarly in ovarian cancer, hormonal influences play a role in angiogenesis. Both LH and FSH levels continue to increase in menopausal women and as such promote disease progression of ovarian cancer due to their contribution in promoting tumor angiogenesis (53). In ovarian cancer, elevated levels of LH promote angiogenesis specifically through the PI3K/Akt-mTOR pathway (54). As previously mentioned, EOC primarily occurs in post-menopausal women, and as such some post-menopausal women are on HRT. Estrogen HRT is known to be a contributing risk factor to the onset of ovarian cancer. This is partially due to the fact that estrogen is known to enhance tumor growth as well as drive angiogenesis, primarily through the mediation of bone marrow-derived cells, endothelial cells and directly downregulating thrombospondin-1 expression (55, 56).

The process of angiogenesis is crucial in the development and progression of solid tumors. Without angiogenesis, tumors would not be able to grow larger than 1-2mm^3^ (57). By initiating angiogenesis, the tumor can stimulate the formation of blood vessels to supply oxygen and nutrients and to facilitate metabolic waste removal (58). To stimulate blood vessel formation, the tumor undergoes an “angiogenic switch” in which pro-angiogenic factors are over-expressed while angiogenesis inhibitors are concomitantly suppressed (59). Angiogenesis does not occur uniformly throughout the solid tumor and most tumors have a mosaic pattern of blood vessels (60). This lack of equal distribution of blood vessels contributes to normoxic regions within the tumor that are highly vascularized allowing oxygen to diffuse to the tumor cells. Typically following the angiogenic switch, the pro-angiogenic stimulus is aggressive, resulting in the rapid formation of tumor vessels (61). As a result of the rapid vascularization, many tumor vessels have altered morphology, with blind-ends, constrictions, shunts and other malformations (62). In addition, tumor vessels typically lack smooth muscle cell coverage and are considered immature (63). As a result of these malformations, tumor vessels are very inefficient in perfusing the tumor, resulting in widespread hypoxia (64). Hypoxic tumor cells can stimulate VEGF expression which activates neighbouring endothelial cells, further increasing angiogenesis within the TME and formation of dysfunctional tumor vessels (65). This reduced vascular perfusion represents a significant impediment to cancer therapy success as the treatment compounds cannot reach the interior of the tumor. Hypoperfusion and the resultant hypoxia is associated with the development of chemoresistance, which is a major problem in ovarian cancer patients (66, 67). Women with EOC that develop chemoresistance have demonstrated a specific angiogenic gene signature which may facilitate therapies targeted in these patients (68).

Tumor vasculature is also important in facilitating tumor metastasis (69). Due to the excessive stimulation by pro-angiogenic factors such as VEGF, tumor vessels are highly fenestrated, with increased space between endothelial cells (70). Due to the increased fenestration, tumor cells can more easily penetrate and enter the vasculature, enhancing metastasis (71). Excessively fenestrated tumor vessels also contribute to fluid extravasation and accumulation in the perivascular space which increases interstitial fluid pressure (IFP) (72). Elevated IFP creates an additional barrier to therapy uptake due to the elevated pressures within the tumor (73). High IFP can also cause the collapse of intratumoral lymphatic vessels, which can obstruct proper lymphatic drainage (74). A combination of hyperpermeable vasculature and lack of lymphatic drainage seen in EOC results in leakage in to the peritoneum through an osmotic effect (75). This contributes to the accumulation of ascites in the abdomen, and the release of tumor cells into the ascites increases metastasis (76).

## Hypoxia as a Feature of Ovarian Cancer Progression

As tumors grow and become less oxygenated and hypoxic, cancer cells develop mechanisms to survive under lower oxygen tension (77). The immediate molecular response to low oxygen is the stabilization of HIFs. HIFs in turn, will activate a number of survival pathways that promote proliferation, angiogenesis and invasion while concurrently inhibiting apoptotic cancer cell death (78, 79). In opposition to this, normal, non-cancerous cells typically respond to hypoxia by undergoing senescence, arresting mitosis and dying by apoptosis if there is DNA damage (80, 81).

Hypoxia is a key inducer of angiogenesis and an activator of the angiogenic switch during tumor development (82). This event is a tip in the balance of pro-angiogenic and anti-angiogenic factors within the TME in response to stimuli, favouring a transition from angiogenic dormancy to a vascularized tumor. HIF-1 is a heterodimer, consisting of a constitutively expressed HIF-1β subunit and a regulatory HIF-1α subunit (83). In response to tissue hypoxia, HIF-1α stabilizes, accumulates and translocates to the nucleus. Once translocated to the nucleus, HIF-1α binds to conserved hypoxia response elements to activate hypoxia-sensitive genes such as VEGF by binding to their promoter regions (84). Culture of ovarian cancer cells under hypoxic conditions results in a concomitant increase in expression of HIF-1α and VEGF (85) and inhibition of HIF-1α results in a significant decrease in VEGF production and tumor angiogenesis (86). HIF-1α also targets and upregulates the stanniocalcin (STC) gene, known for being an anti-hypercalcemic glycoprotein hormone, although not much research has been focused on STC in ovarian cancer tumorigenesis (87). STC1 overexpression has been linked with increased proliferation, migration and colony formation in human ovarian cancer cell lines as well as increased expression of cell cycle regulation proteins and increased expression of anti-apoptotic proteins, hindering apoptosis (88). Under hypoxic conditions, HIF-1α regulates increased STC2 expression to facilitate increased ovarian cancer tumor cell proliferation (89). Both STC1 and STC2 promote epithelial to mesenchymal transition in hypoxic ovarian cancer cells and contribute to their invasion and metastasis as well (90, 91). Additionally, estrogen and progestin can directly regulate HIF-1α through the PI3K/Akt-mTOR signaling pathway and contribute to tumor metastasis in ovarian cancer (92). In EOC, HIF-1α expression is upregulated and its increased expression is linked to poor survival (93). HIF-2α on the other hand dimerizes with HIF-1β and binds to hypoxia response elements similarly to HIF-1α. In EOC, women with advanced disease, either stage 3 or 4, have a specific HIF profile characteristic of elevated nuclear expression of HIF-1α and elevated cytoplasmic HIF-2α expression, and this specific profile is associated with a poor prognostic outcome (94).

Several hypoxia-mediated changes within the TME may explain the link between poor prognosis and HIF-1α expression. Hypoxia contributes to the selection and activation of an ovarian cancer stem cell niche and cancer stem cells (CSCs) appear to favour hypoxic sites within tumors (95). CSCs are undifferentiated, or less-differentiated cells that drive tumorigenesis and give rise to the large population of cells that comprise majority of the tumor (96). CSCs can create a significant therapeutic challenge as they can evade and resist chemotherapy due to their stem cell qualities. CSCs are quiescent, or very slowly cycling, which renders them resistant to therapies that target rapidly dividing cells (97). CSCs also upregulate survival signaling pathways, making them harder to kill with conventional cytotoxic therapy (98). CSCs act as a cell reservoir, and can be a major contributor to cancer recurrence (99), including that seen in ovarian cancer (100). It has been suggested that the correlation of poor patient outcome with hypoxia may be related to the enhanced presence of CSCs (101). The Notch, Wnt and Hedgehog pathways are important in the maintenance, self-renewal and resistance of CSCs (102, 103). In ovarian cancer, HIF-1α activates Notch1 signaling, which increases the activity of the Sox2 promoter, creating a CSC phenotype and drives drug resistance in ovarian cancer stem cells (104). In ovarian cancer cells, exposure to hypoxia increases expression of CD44, CD133, Oct3/4 and Sox2, which are known markers of ovarian CSCs (105). The other isoform of HIF, HIF-2α, also targets Notch, Oct4 and Sox2 (106), suggesting that HIF-2α is also important in maintaining stemness in the hypoxic TME. HIF-1α also upregulates the expression of Sirtuin type 1, which is known to promote CSC-like features in ovarian cancer cells (107).

A unique feature of peritoneal tumors, including ovarian cancer, is the presence of ascites fluid. This complex mixture of soluble factors accumulates due to leaky tumor vasculature as well as disrupted lymphatic patency (108). Given that ascites worsens tumor access to oxygen, ascites accumulation promotes the negative responses of the tumor to low oxygen (109). In addition, the flow of ascites fluid current dictates the direction of EOC secondary tumor dissemination within the abdominal cavity (108). Malignant ascites also contains factors which induce immunosuppression and enhance survival – creating an ideal environment for tumor dissemination to other abdominal organs by evading the immune system.

## Hypoxia Alters the Immune Environment in Ovarian Cancer

The tumor immune microenvironment (TIME), which encompasses not only malignant transformed cells, but also normal cells such as epithelial cells, fibroblasts, endothelial cells, muscle cells and immune cells in EOC has been described as ‘highly permissive’ to tumor growth, metastasis and therapy resistance (110–112). While tumor subtypes such as melanoma and lung cancer present with high levels of interferon and T cell infiltrates and are thus excellent candidates for immunotherapy, the TIME in ovarian tumors contains a distinct suppressive phenotype populated by immature myeloid cells, anergic T cells and T regulatory (Treg) cells (113). As previously discussed, ascites fluid is rich in immunosuppressive cytokines and therefore drives production of these suppressive cells and acts as an ideal conduit in the spread of tumor nodules to other organs (114). Ascites also contains significantly higher proportions of T cells expressing checkpoints such as LAG‐3+, PD‐1+, TIM+ and CTLA‐4+ compared to peripheral blood (115).

Low oxygen partial pressure within the tumor activates hypoxia-dependent signaling, where HIFs are stabilized. HIF-1 maintains elevated myeloid-derived suppressor cell levels and regulates their function and maturation (116). Myeloid-derived suppressor cells in turn lead to the production of immunosuppressive cytokines, of which TGF-β, IL-6 and IL-8 contribute to high immunosuppression in advanced ovarian cancer (117). Hypoxic areas also attract and polarize M2 type tumor-associated macrophages (118). The function of this subtype of macrophages is to promote tissue repair, which involves immune tolerance and modulation (119). M2 macrophages are present in higher proportions in advanced stages of ovarian cancer compared to early stages of the disease (120), indicating that they may be linked to disease progression. In addition, a higher M2/M1 tumor-associated macrophage ratio is an indicator of positive prognosis in EOC and has been reliable in predicting patient survival time in other cancers (121). Hypoxia also upregulates CCL28, a chemokine for Treg cells, in a HIF-dependent manner (122). Tregs in turn challenge anti-tumor immune responses by downregulating effector T cells (123). A high CD8+/Treg ratio is a significant predictor of prognosis in ovarian cancer (124). As the most potent antigen-presenting cell, dendritic cells (DCs) play a major role in tumor immunosurveillance. Prolonged exposure of DCs to hypoxia leads to cell death of these antigen presenting cells – a process that can be prevented by inhibiting HIF-1α (125). Additionally, expression of HIF-1α diminishes the ability of DCs to produce IL-12 – an important cytokine for the development of cytotoxic T cells (126). STC1 has been shown to interact with and decrease membrane exposure of calreticulin, impairing phagocytic responses of antigen presenting cells, including DCs and macrophages, to ultimately inhibit antigen presentation to facilitate T cell activation (127). Additionally, STC1 inhibits macrophage infiltration, further hindering an immune response against the tumor (128). Altogether, while malignant cells thrive in the absence of oxygen, immune cells which would ideally produce an anti-tumor response against tumor associated antigens, frequently become anergic or die in response to this environment (129). This system imbalance creates a pro-tumorigenic environment and hinders patient response to immunotherapies.

The fundamental basis of successful immunotherapy to treat cancer is positive immunogenicity of the specific tumor subtype, which considers presence of tumor associated antigens and effective presentation of these antigens. The immunosuppressive TIME as well as low mutation rate, which impairs neo-antigen formation, challenges the use of immune-based therapies in ovarian cancer (130–132). Findings have demonstrated that ovarian tumors are typically “cold” meaning that they lack cytotoxic T cell infiltration. Goode et al. demonstrated a distinct dose-response relationship between the number of CD8+ infiltrates and patient survival time in high-grade serous ovarian cancer (133). The prognostic impact of tumor infiltrating lymphocytes (TILs) trafficking suggests that the factors which regulate TIL infiltration are vital when improving current therapies or seeking new targets. In EOC, T cells face physical barriers such as vascular access, which impede their access to tumor cells. Abnormal vasculature within EOC tumors leads to downstream hypoxia, which has been named a common biological determinant of immune suppression in solid tumors (134). Indeed, mono-immunotherapy in ovarian cancer has yielded modest results (135, 136). Battaglia et al. found that assessment of immune status prior to treatment with a CA-125 targeted monoclonal antibody may predict treatment sensitivity (137). EOC patients vary with respect to levels of TILs and individual tumor samples have pronounced heterogeneity in immune profile (138, 139). In light of these findings, more recent studies into EOC immunotherapy focus on personalized and combination strategies. The anti-angiogenic compound Avastin has shown promising results in combination with immunotherapy in light of reducing molecules such as VEGF, thereby enhancing inflammation. Between patients, previous exposure to chemotherapy, disease histotype and ascites profile are each indicators of immune therapy success (133, 140, 141). These findings illustrate that immunotherapy may be a successful treatment strategy for EOC in the future, although the tumor microenvironment must be considered in therapy design.

## The Impact of Hypoxia on Therapy Resistance

Hypoxia has been associated with the development of chemoresistance through a variety of mechanisms. One of the mechanisms by which hypoxia induces chemoresistance is through alteration of cancer cell metabolism. In response to hypoxia, ovarian cancer cells undergo a metabolic switch, with changes in the glycolytic pathway that promotes resistance to carboplatin (142–144). As ovarian tumors become hypoxic, there is an upregulation of glycolytic enzymes to metabolize glucose, resulting in the formation of lactate (145). The reduction in pH from lactate accumulation inhibits the efficacy of chemotherapy agents (144). HIF-1α appears to be a central regulator of cellular metabolism. HIF-1α regulates many of the enzymes involved in glucose catabolism and regulates lactate production through activation of lactate dehydrogenase A and the lactate transporter MCT4 (146). As HIF-1α drives cells toward anaerobic glycolysis, it supports the metabolic switch seen in ovarian cancer cells. Inhibition of HIF-1α is a strategy that has been employed to redirect cells to oxidative phosphorylation, resulting in the production of cytotoxic levels of reactive oxygen species and cancer cell apoptosis (147). Specifically targeting HIF-1α through antisense as an anti-cancer strategy has shown efficacy in xenograft models of ovarian cancer (148). HIF-1α also contributes to the development of platinum resistance through induction of cancer cell autophagy (149). Autophagy is a process that assists in maintaining cell viability during times of stress. Under stressful conditions, autophagy is rapidly activated to reduce cellular growth and increase catabolic lysis of unnecessary proteins and organelles. However, persistent or excessive autophagy can lead to the induction of cell death (150). Activation of autophagy in ovarian cancer is associated with the development of chemoresistance through the induction of the MAPK/ERK survival pathways (151). HIF-1α initiates autophagy in ovarian cancer cells exposed to hypoxia, and these cells develop resistance to cisplatin-induced apoptosis (149). In the challenging, hypoxic and nutrient-poor TME, autophagy may be an important mechanism employed by tumor cells to survive and develop chemotherapy resistance. HIF-1α has also been found to directly induce expression of the STC1 gene, producing STC in human ovarian cancer cell lines (152). STC has been shown to regulate a number of oncogenic effects in different tumor subtypes such as triggering angiogenesis through upregulation of VEGF in gastric cancer (153), as well as exacerbating chemoresistance, invasion and metastasis in breast cancer (154–156). Although the mechanistic details behind STC in ovarian cancer have not been heavily studied, its expression is highest in the ovaries, particularly during pregnancy and lactation (157). In addition, dysregulated levels of STC have been linked to poor outcome in patients, lending merit to further investigations into its functions (88).

Hypoxia also regulates the expression and function of a multitude of microRNAs (miRNAs). MiRNAs are small, approximately 19-25 nucleotides endogenous non-coding RNAs that regulate gene expression (158) in humans, as well as a wide array of organisms (159, 160). MiRNAs have been implicated in the onset, progression and therapy resistance in ovarian cancer (161). Hypoxia is known to regulate the expression of several miRNAs in ovarian cancer. Hypoxia in ovarian cancer is associated with altered levels of circulating miRNAs and these expression profiles are associated with the risk of developing ovarian cancer. In one study, 36 miRNAs were overexpressed, and 101 miRNAs were downregulated in women at high-risk of ovarian cancer, compared to those with low risk (162). HIF-1α hypoxia-induced overexpression of miR-210 in EOC has been shown to promote proliferation and migration while inhibiting apoptosis of EOC *in vitro* (163, 164). Interestingly, deletion of miR-210 is also associated with dysregulated cell cycle and progression of ovarian cancer (165), suggesting that miRNA effects may be context-specific. In addition to regulating tumorigenic processes, hypoxia-induced miRNA expression has been implicated in the acquisition of drug resistance in ovarian cancer. Under hypoxic conditions, the HIF-1α pathway increases expression of miR-27a and is associated with the development of paclitaxel resistance through downregulation of the apoptosis-related protein APAF1 (166). Hypoxia has also been implicated in drug resistance in patients with high HIF-1α expression through upregulation of expression of miR-223 and activation of the PTEN-PI3K/Akt-mTOR pathway, resulting in multi-drug resistance and disease recurrence (167). MiRNA expression in response to hypoxia in EOC appears to regulate numerous important tumorigenic processes and response to therapy, although the mechanisms involved are still largely unclear.

## Reversal of Hypoxia as an Approach to Personalized Therapy

HIF-1 expression alone is not an indicative prognostic marker onRESA the disease progression in ovarian cancer, however, overexpression of HIF-1 in combination with the presence of non-functional p53 is associated with a more aggressive phenotype and a poorer prognosis (168). A gain-of-function mutation of the p53 gene is characteristic of the most common subtype of EOC, high-grade serous ovarian cancer (169), therefore ovarian cancer patients with a high HIF-1 expression profile may benefit from therapies directly targeting HIF-1. There are many novel therapeutic agents targeting HIF-1 activity that are currently in clinical trials. These therapeutic agents are designed to directly inhibit HIF-1 activity through a multitude of various targets including mTOR (170), COX2 (171), epidermal growth factor receptor EGFR (172), heat shock protein 90 (173), topoisomerase I (174) or at the post-transcriptional level (175). Herceptin is a monoclonal antibody that specifically targets human epidermal growth factor receptor 2 (HER2) and is currently an FDA approved therapy for the treatment of early and late stage breast cancer patients with HER2 overexpression (176). The mechanisms of action of Herceptin are not fully understood, however, many outcomes of treatment with Herceptin have been observed. Herceptin prevents HER2 and Src tyrosine kinase from clustering, inhibiting activation of PI3K/Akt-mTOR and MAPK/ERK signaling pathways (177). Herceptin also induces tumor cell apoptosis and cell cycle G1 arrest (178, 179). Overexpression of HER2 in tumor cells is known to be affiliated with increased VEGF expression and increased angiogenesis (180). Herceptin decreases expression of pro-angiogenic factors and subsequently increases expression of anti-angiogenic factors (181) as well as reduces endothelial cell migration (182). In ovarian cancer, HER2 is expressed in up to 66% of EOC cases and is associated with a poorer prognosis (183, 184). While not much research has been focused on the use of Herceptin in treating ovarian cancer, there may be a subset of ovarian cancer patients with HER2 overexpression that may benefit from Herceptin incorporated into their treatment plan, but this needs to be explored in greater detail. Alternatively, targeting downstream effects of HIF-1α are also potential therapeutic avenues. STC1 and STC2 are upregulated and promote tumorigenesis and disease progression in a HIF dependent manner as previously discussed and may be a future target of interest. Recent studies targeting STC1 with a vector expressing a suicide gene under a STC promoter inhibited and arrested cell growth in lung cancer cell lines (185) although further research is still required before its potential clinical use.

Hypoxia-activated prodrugs were designed to specifically target the hypoxic tumor cells within a solid tumor. Their mechanism of action work by their enzymatic reduction to reactive oxygen species under hypoxic conditions, with capacity to re-oxidize in oxygenated environments. In pre-clinical and clinical settings, hypoxia activated prodrugs have shown promising efficacy in successfully attacking hypoxic cells of solid tumors (186–188). This approach has been more efficacious when combined with chemotherapy and radiotherapy (189, 190), as well as anti-angiogenic therapies (191–193). Many solid tumor cancers, including EOC, are known to have overexpressed folate receptors on the tumor cell surfaces (194). Folate-based redox-responsive nanoparticles (NPs) and designed to target and bind to the folate receptor with high affinity. NPs are capable of releasing therapies solely in the presence of hypoxia due to their design of being cleaved only in the absence of oxygen (195). Folate acid-tagged NPs can be loaded with cancer therapies, such as chemotherapy and immunotherapy and rapidly release these drugs under hypoxic conditions compared to normoxic regions (195–198). Anti-angiogenic application of NPs are also being employed through use of gold, silver and silicate-based NPs. Their mechanism of action work to inhibit VEGF and other pro-angiogenic factors as well as induce production of reactive oxygen species causing vessel constriction and thereby inhibiting proliferation and migration of endothelial cells, which ultimately halts tumor cell growth (199–202).

Anti-angiogenic therapies can normalize tumor vasculature, uniformly increase oxygen delivery and reverse the hypoxic areas of the tumor. This allows for better uptake and distribution of therapeutic drugs to the tumor (203). Many anti-angiogenic therapies work by inhibiting VEGF, either by directly binding to VEGF or inhibiting the VEGF receptor. By inhibiting only angiogenesis, these therapies do not interfere with normal pre-existing vasculature in patients. The anti-angiogenic therapy Avastin is an FDA approved monoclonal antibody used in combination with chemotherapy primarily when recurrence of EOC occurs (204). This includes patients that are both platinum-sensitive and platinum-resistant (205). Avastin works by binding to VEGF with high specificity and therefore prevents it from binding to the VEGF receptor. Combination of Avastin with chemotherapy at the first onset of platinum resistance in ovarian cancer patients demonstrates the greatest outcome for overall patient survival, compared to Avastin or chemotherapy alone (206). Additionally, anti-angiogenic therapies when combined with NPs in platinum resistant patients are also being investigated for clinical safety and their potential efficacy (207). Other anti-angiogenic therapies targeting the VEGF receptor show similar efficacy, especially when used in combination with other cancer therapies, including chemotherapy (208, 209) and immune checkpoint inhibitors (210). More recently, the combination of Avastin and the poly (ADP-ribose) polymerase (PARP) inhibitor, olaparib, have been FDA approved for combination use in patients who responded to chemotherapy or in patients that carry a BRCA1 or BRCA2 mutation (211). Due to the nature of its use, rapid tumor resistance to Avastin often occurs due to upregulation and reliance on pathways other than VEGF by the tumor (212, 213). As such other new anti-angiogenic therapies are being developed to overcome this issue and demonstrate promising potential. Treatment with 3TSR, a novel compound derived from the anti-angiogenic regions of thrombospondin-1, has demonstrated its ability to reduce primary ovarian tumor size, occurrence of metastatic tumors and decrease abdominal ascites accumulation in a murine EOC model when used in combination with chemotherapy (214) and oncolytic viruses (215). Neferine, a bisbenzylisoquinoline alkaloid derivative from lotus seed embryos, demonstrates anti-angiogenic properties in chemoresistant EOC by inducing autophagy and inhibiting macrophage maturation (216).

Estrogen receptors are specifically located on smooth muscle pericytes within blood vessels and estrogen is known to inhibit proliferation of vascular smooth muscle cells (217, 218). Additionally, estrogen is known to be anti-inflammatory and vasoprotective in young women compared to its pro-inflammatory and vasotoxic effects in older women, partially due to altered estrogen signaling pathways with age (219). Tamoxifen, a selective estrogen receptor modulator hormonal therapy, is known to exhibit its anti-angiogenic properties by inhibiting platelet activation (220). Although this hasn’t been studied much in relation to cancer, the presence of estrogen receptors on pericytes and the hormonal status of a woman (menopausal with or without HRT) may result in some patients being less responsive to anti-angiogenic vessel normalization therapies. The addition of HRT may aid in increased efficacy of anti-angiogenic therapies, and this should be taken into consideration when designing a treatment plan for patients with hormone-dependent cancers.

Reversing hypoxia within the TME will not directly contribute to tumor cell death, however, we can utilize this as an advantage to create more personalized combinational therapeutic approaches. The presence of hypoxia and poor tumor vasculature is characteristic to many solid tumors including EOC (221). In light of this, women with ovarian cancer may benefit from initial therapies that normalize the tumor vasculature and reverse a hypoxia followed by administration of a more specific therapy, such as chemotherapy, a PARP inhibitor or oncolytic virus, based on a patient-by-patient basis. By normalizing the tumor vasculature first, we may be able to increase uptake of these therapies by the tumor, have better distribution of therapies throughout the tumor cells and ultimately reduce chemoresistance and increase apoptotic cell death of the entire tumor. This will ultimately lead to more efficacious treatments and increased patient survival.

## Conclusion

Hypoxia-induced changes in the endocrine environment and immune status of the tumor play an influential role in promoting tumorigenesis in ovarian cancer. By understanding the changes that occur in the ovarian TME, we can develop novel therapies that target these changes to improve efficacy and reduce therapy resistance. An approach to personalized treatment strategies on a patient-by-patient basis may ultimately improve the way we treat women with ovarian cancer.

## Author Contributions

The article was conceptualized by JP. MP, KM, CJ, and JP contributed to the writing and editing of the review. All authors contributed to the article and approved the submitted version.

## Conflict of Interest

The authors declare that the research was conducted in the absence of any commercial or financial relationships that could be construed as a potential conflict of interest.

## Publisher’s Note

All claims expressed in this article are solely those of the authors and do not necessarily represent those of their affiliated organizations, or those of the publisher, the editors and the reviewers. Any product that may be evaluated in this article, or claim that may be made by its manufacturer, is not guaranteed or endorsed by the publisher.

## Abbreviations

CSC, Cancer Stem Cell; DC, Dendritic Cell; EOC, Epithelial Ovarian Cancer; FSH, Follicle Stimulating Hormone; GnRH, Gonadotropin-Releasing Hormone; hCG, Human Chorionic Gonadotrophin; HER2, Human Epidermal Growth Factor Receptor 2; HIF, Hypoxia Inducible Factor; HRT, Hormone Replacement Therapy; LH, Luteinizing Hormone; NP, Nanoparticle; OSE, Ovarian Surface Epithelium; PARP, Poly (ADP-ribose) Polymerase; STC, Stanniocalcin; TIME, Tumor Immune Microenvironment; TME, Tumor Microenvironment; Treg, T Regulatory Cell; VEGF, Vascular Endothelial Growth Factor.
